# What are the psychometric properties of a menstrual hygiene management scale: a community-based cross-sectional study

**DOI:** 10.1186/s12889-020-08627-3

**Published:** 2020-04-19

**Authors:** Astha Ramaiya, Suruchi Sood

**Affiliations:** 1 Department of Population, Family and Reproductive Health, Johns Hopkins University, Baltimore, MD USA; 2 Department of Community Health and Prevention, Drexel University, Philadelphia, PA USA

**Keywords:** Menstrual health and hygiene management, Menstrual hygiene management, Psychometric properties, Hygiene, Health

## Abstract

**Background:**

The last decade has highlighted how menstrual hygiene management (MHM) is a public health issue because of its link to health, education, social justice and human rights. However, measurement of MHM has not been validated across different studies. The objective of this manuscript was to test the psychometric properties of a MHM scale.

**Methods:**

An embedded mixed-method design was utilized. The girls (age 12–19) were from three districts of Uttar Pradesh (Mirzapur, Jaunpur and Sonebhadra), India. A total of 2212 girls participated in the structured questionnaire. Trained interviewers collected the data on tablets using computer assisted personal interviewing. A total of 36 FGDs were conducted among 309 girls between. Trained moderators collected the data. Factor analysis and thematic analysis was conducted to analyze and triangulate the data.

**Results:**

More than 90% of the girls were from a marginalized caste. Overall, 28% of the girls practiced all six MHM behaviors adequately. The factor analysis found five separate constructs corresponding to menstrual health and hygiene management (MHHM) with a variation of 84% and eigenvalue of 1.7. Preparation of clean absorbent, storage of clean absorbent, frequency of changing and disposal loaded separately, corresponding to menstrual health. Privacy to change and hygiene loaded together (eigenvalue 0.91 each), corresponding to hygiene management. An underlying theme from the FGD was menstruation as a taboo and lack of privacy for changing the absorbent.

**Conclusion:**

MHM is multi-dimensional construct comprising of behaviors which were time-bound by menstruation (menstrual health) and behaviors not time-bound by menstruation (hygiene management). Based on these results, the author recommends that MHHM is used as an acronym in the future and proposes a revised definition for MHHM.

## Background

The last decade has highlighted how menstrual hygiene management (MHM) is a public health issue because of its link to health, education, social justice and human rights [1, 2]. MHM is defined as the “use of clean menstrual management material to absorb or collect blood that can be changed in privacy as often as necessary for the duration of the menstruation period, using soap and water for washing the body as required and having access to facilities to dispose of used menstrual management materials” [3, 4].

Initially, MHM was brought to the public health agenda in order to decrease the gender inequality in education and to keep adolescent girls at school. Adolescent girls around the world have reported feeling ashamed and afraid once they start menarche. This feeling lowers their self-confidence and decreases their decision making power regarding sexual and reproductive health [5]. Furthermore, research in South Asia has shown that schools’ lack of adequate water and sanitation facilities affects the ability of adolescent girls to meet their MHM needs with dignity [6–8].

Measurement of MHM has not been validated across different studies [2]. A total of eight scientific studies have made a composite index for MHM, five of which were conducted in India [9–16]. Other scientific publications have not constructed a scale but instead examine individual behaviors as separate variables to assess MHM [17–25].

**Fig. 1:**
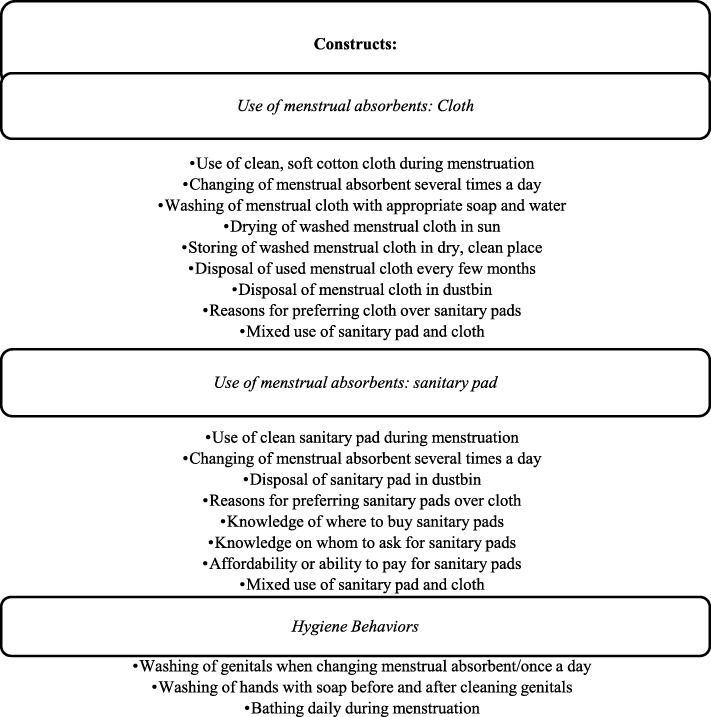
Framework for Measuring Menstrual Behaviors Adapted from Muralidharan [26]

A new MHM framework *(*Fig. 1*)* developed by Muralidharan outlines components highlighted below [26]. Some adaptations have been made on this framework by operationalizing only “behaviors”, since the purpose of this research is to create and measure the psychometric properties of an MHM behavioral scale.

Figure 1 and the MHM definition demonstrates how the construct can be operationalized. The definition outlines a total of six indicators:

- Preparation of clean absorbent: What type of absorbent is used, where is it obtained from and is it clean? If a cloth is reused, then is it washed with soap or disinfectant & water, dried completely in the sun and used exclusively?
- Storage of clean absorbent: Is the absorbent stored in a clean place for example, with other clothes?
- Ability to change absorbent in privacy: Is there privacy at home and school to maintain hygienic care?
- Frequency of changing: How many times is the absorbent changed in a day?
- Disposal: Where and how is the absorbent disposed?
- Hygiene: Are there facilities to take a bath, wash hands with soap and water to maintain personal hygiene during menstruation?

In the literature, different scales are measuring different indicators of MHM with different items, yet most scales have not measured all indicators. Additionally, most studies have assessed the indicators differently and almost none have described the psychometric properties of their scale. In one study, internal consistency of the scale was conducted but it was only during the pre-testing stage among 20 adolescent girls (Cronbach’s alpha = 0.9) [14]. A total of five studies within India have made a composite of MHM behaviors whereas seven studies have assessed individual MHM behaviors [10–14, 19–25].

The objective of this manuscript is to develop and test a MHM scale using the Joint Monitoring Program definition, current literature and the constructs developed by Muralidharan; to operationalize and carry out psychometric testing of a MHM scale among adolescent girls in three districts of rural Uttar Pradesh [3, 26].

## Methods

### Data sources/measurement of MHM scale items

To create a robust MHM scale, several types of validity were examined, including content validity and construct validity. Appendix 1 outlines the justification for the different validity and reliability tests for this study.

In order to measure content validity, first, the MHM definition developed by the joint monitoring program was used; second, the scale was operationalized based on other scales within literature and on Muralidharan’s framework [3, 26]. The literature was perused to understand which indicators of MHM had been measured. The results obtained from this literature review helped in operationalizing the constructs outlined within the joint monitoring program definition and Muralidharan’s framework.

Another form of content validity involved consultation with an expert committee to provide their thoughts and assessment on each item of the MHM scale. All the questions being considered for the MHM scale were provided to the experts and they were asked to respond on a 1–3 scale, with 1 representing a non-essential item, 2 representing a useful but non-essential item and 3 representing an essential item [27]. Item validity was determined: 1) If the item validity was > 0.79, it was considered appropriate; 2) item validity between 0.7–0.79, needed revision and 3) if the item validity was < 0.7, a recommendation was made to eliminate the item [27]. A total of six experts within the field of MHM were asked to provide their feedback on the scale. Overall, 81.25% of the questions were considered appropriate by the six experts. Three questions which had an eliminate recommendation were retained in the analysis since data from the formative research and pre-testing, showed this was a valid response category as a MHM behavior.

One way that construct validity was examined, was by piloting a MHM scale in October 2016 (Appendix 2*)*. Data from 450 adolescent girls was included in the exploratory factor analysis. The analysis demonstrated two factors (eigenvalue of 1.2 and 1.0) with the first factor including storage of clean absorbent and hygiene (factor loadings of 0.7 and 0.9) and the second factor including preparation of clean absorbent, frequency of changing and disposal (factor loadings of 0.3, 0.4 and 0.9, respectively). Based on the feedback from the pilot, the current scale was created.

The literature review, concurrent monitoring data and expert interviews were helpful to construct the current scale. The current scale was pretested. A majority of the data to answer this research question comes from the quantitative component. Relevant qualitative data was also collected and used to complement and explain the quantitative findings.

### Study design & setting

This study was an embedded mixed method cross-sectional study, which predominantly included results from a household structured questionnaire with triangulation from focus group discussions (FGDs) in three districts (Mirzapur, Jaunpur and Sonebhadra) of Uttar Pradesh from December 2017–January 2018.

### Sampling frame, participants and sample size

Participants for both the structured questionnaire and FGDs were selected by random sampling, stratified by religion, caste, education and age. A total of 2212 adolescent girls were selected across 240 villages for the structured questionnaire and 309 adolescent girls were selected for the FGDs. The sample size calculation has been reported in another paper [28].

### Data collection

The structured questionnaire and FGDs were translated into Hindi and pre-tested to decrease information bias. A participatory FGD activity called “Day in the life of” was conducted. The structured questionnaire was completed on tablets using computer assisted personal interviewing by trained data collectors to increase inter-rater reliability and reduce non-response bias [29, 30].

### Tool descriptio

This section outlines how the validity of MHM behaviors was operationalized and measured quantitatively and qualitatively.

#### Quantitative tool description

The validity of a six-question quantitative MHM scale was being tested in this manuscript. Table 1 outlines how preparation of clean absorbent was operationalized. Preparation of the clean absorbent had between 1 and 4 questions based on the type of absorbent used. In order to adequately account for preparation of clean cotton cloth, adolescent girls were asked how often they washed the cloth before they used it, how they washed their menstrual cloth, how they dried the cloth and if they used the cloth exclusively.

**Table 1:** Menstrual Hygiene Management Scale (Preparation of Clean Absorbent)

Question Number	Indicator	Question	Responses
1	Preparation of clean absorbent	What type of menstruation absorbent do you use? *Multiple response*	1. new cotton cloth every time I change 2. Old cotton cloth 3. Any other synthetic cloth 4. Disposable sanitary pad 99.Any other (specify) ______________
1.1	Preparation of clean absorbent – Old cotton cloth (option 2)	Do you wash the cloth before you use it to absorb menstrual blood?	1. Always 2. Mostly 3. Sometimes/occasionally 4. Rarely Never
1.2	Preparation of clean absorbent – Old cotton cloth (option 2)	How do you wash your menstrual cloth? *Multiple response*	1. Water only 2. Soap and water 3. Any other disinfectant after washing with soap and water 4. Hot water 99.. Any other (specify)______
1.3	Preparation of clean absorbent – Old cotton cloth (option 2)	Where do you dry your menstrual cloth? *Multiple response*	1. In the shade outside 2. In the shade inside 3. In the sunlight outside 4. I hide it 99. Any other (specify) ________
1.4	Preparation of clean absorbent – Old cotton cloth (option 2)	Do you use menstrual cloth exclusively or share with other female members of the family?	1. Exclusively use 2. Shared with others

Storage of clean absorbent was asked as “how do you store your menstrual absorbent?” Multiple responses could be provided with the following response choices: 1. Store the absorbent in a hidden or concealed place; 2. Store the washed menstrual cloth along with other clothes of daily wear; 3. Bag; 4.Does not store, takes new one every month; 5. Store in a safe clean place; 6. In cupboard; 7.In a bag, hang it; 8.In a plastic bag; 9. Polythene bag; 10.Wrapped in paper, kept in bag; 99. Any other (specify).

Ability to change absorbent in privacy was asked as: “during menstruation where do you change your absorbent?” Response choices included: 1. In a private bath area; 2. In a private toilet; 3. Behind a curtain; 4. Behind a temporary structure; 5. Inside house; 6. Any other (specify).

Frequency of changing was operationalized as: “How many times do you change your menstrual absorbent everyday?”. Adolescent girls provided a numeric response to the question.

Disposal was operationalized as “How do you ultimately dispose of your used menstrual absorbent?” Multiple responses were permitted for this question. Response choices included: 1. Bury it in the soil in the field; 2. Throw it in the bush; 3. Burn the absorbent; 4. Hide under a stone; 5. Store and then take it to the school toilet incinerator; 6. Dustbin; 7. Garbage lot; 8. Bury in a pit; 9. Pond water body; 10. Gutter; 11. Wash it; 99. Any other (specify).

Hygiene was operationalized with 8 questions to avoid double barreled questions (Table 2). For the analysis it was necessary to understand if these behaviors were done normally and during menstruation in order to determine if there was a difference in behavior during menstruation.

**Table 2:** Menstrual Hygiene Management Scale (Hygiene)

Question Number	Indicator	Question	Responses
6.1	Hygiene	Is there a separate bathing place at home?	1. Yes 2. No
6.2	Hygiene	Do you normally use it for bathing?	1. Yes 2. No
6.3	Hygiene	Do you use the bathing area at home during menstruation?	1. Yes 2. No 3. Do not bathe during menstruation
6.4	Hygiene	Do you normally take a bath with soap and water?	1. Yes 2. No
6.6	Hygiene	During menstruation do you take a bath daily with soap and water?	1. Yes 2. No
6.7	Hygiene	During menstruation, do you always wash your hands after changing your menstrual absorbent?	1. Yes 2. No
6.8	Hygiene	How do you wash your hands after changing your menstrual absorbent?	1. With only water 2. With soap and water 3. With ash and water 4. With mud and water 5. Others (specify)

Appendix 3 outlines the entire tool used to measure MHM in this study and was created by the first author of this manuscript.

Table 3 outlines how preparation of clean absorbent, storage of clean absorbent and disposal were coded as adequate [2], semi-adequate [1] and inadequate (0). Ability to change absorbent in privacy, frequency of changing and hygiene were coded dichotomously. For all six behaviors, adequate was considered the gold standard practice. Ability to change absorbent in privacy was coded as adequate if adolescent girls said in a private bath area or private toilet. Adequate frequency of changing was three or more times a day. Hygiene was coded as adequate if adolescent girls said they had a separate bathing place at home, used it normally and during menstruation for bathing, took a bath with soap and water regularly and during menstruation; and washed their hands with soap and water after changing the menstrual absorbent. All other responses for these three indicators were coded as inadequate.

**Table 3:** Menstrual Hygiene Management Variable Coding as Adequate, Semi-Adequate or Inadequate

Indicator	Adequate MHM (2)	Semi adequate MHM (1)	Inadequate MHM (0)
Preparation of clean absorbent	Preparation of absorbent was coded adequate if the adolescent girls used new cotton cloth every time they changed or a disposable sanitary pad OR If the adolescent girls used old cotton cloth, they had to wash their cloth with soap and water or any other disinfectant after using soap and water; dry the cloth in the sunlight outside; and exclusively use the menstrual cloth.	If adolescent girls practiced both adequate and inadequate preparation of absorbent. I.e. used sanitary pad and used old cotton cloth which was not dried in the sunlight.	If synthetic cloth was used or if new cotton cloth, disposable sanitary pad was not selected, and old cotton cloth was not practiced adequately.
Storage of clean absorbent	Storage of absorbent was coded as adequate if adolescent girls stored the absorbent along with other clothes of daily wear, in a safe clean place, in a bag or in the cupboard. OR If the adolescent girls stated they used sanitary pad and new cotton cloth every time they changed, not storing was coded as adequate.	If adolescent girls practiced both adequate and inadequate storage of absorbent. I.e. stored the absorbent with other clothes of daily wear, but also stored it in a hidden or concealed place.	If adolescent girls stored the absorbent in a hidden or concealed place.
Disposal	Disposal was coded as adequate if the absorbent was burnt, stored and taken to the school incinerator or buried in a pit.	If adolescent girls practiced both adequate and inadequate disposal of absorbent. I.e. burnt the absorbent and buried it in the soil in the field.	Disposal was inadequate if the absorbent was buried it in the soil in the field, thrown it in the bush, hid under the stone, thrown it in a dustbin/garbage lot, thrown it in a pond or gutter or washed it.

### Qualitative tool description

A participatory FGD activity labelled as “Day in the life of” combined both narrative and visual methods to understand what a typical day looks like for a menstrual absorbent from the perspective of an adolescent girl. Adolescent girls were split into two groups and provided menstrual cloth and a sanitary pad to serve as visuals. Both groups were then given a sheet of paper, where they recorded responses to questions, while imagining they were a menstrual absorbent (cloth and sanitary pad) to understand preparation, storage/use, disposal and hygiene behaviors. Both groups were given the same set of questions, with the cloth group being asked about washing the cloth with soap and water and drying it completely in the sun prior to re-use. The adolescent girls who were responding to questions from the perspective of a sanitary pad were not asked these specific questions on reuse. The two groups of adolescent girls then came together to discuss and compare their answers to determine ways in which cloth and sanitary pad use was similar and different from one another. The tool for “Day in the life of” is attached in Appendix 4*.*

### Analysis

This section outlines the methods used to analyze the quantitative and qualitative data:

#### Quantitative analysis

The quantitative data was first analyzed for socio-demographics and MHM behavior characteristics of the population. Second, an exploratory factor analysis was conducted to assess the dimensionality of the MHM scale during the endline.

The following socio-demographic, socio-economic variables were collected as part of this study: religion, caste, marital status, age, education, type of house and duration since menarche.

To test the psychometric properties of the MHM scale, a polychoric correlation factor analysis was conducted because the indicators were coded as an ordinal scale (0,1 or 2) or dichotomously (0 or 2). If the eigenvalue was ≥1, the factor was retained.

There were no missing values for any of the six indicators. All analysis was conducted on Stata 14.0 [31].

#### Qualitative analysis

For the “day in the life of” activity, each question had its own list of responses for sanitary pad and cloth. Deductive coding was used to create themes for analysis. Themes included what the absorbent is made of, place where it is obtained, storage, how it is used, place where it is changed, frequency of changing, how it is carried around, is it reused, cleaning and drying of cloth and disposal. The responses for cloth and sanitary pad were kept separate. A narrative analysis was written stratified by sanitary pad and cloth.

## Results

### Univariate analysis of socio-demographics, socio-economic and MHM behaviors

Socio-demographics (district, religion, age, education, caste and marital status), socio-economic (type of house) factors and MHM behaviors of adolescent girls were assessed. Table 4 shows that there was an equal distribution of the respondents across districts (36.71% in Jaunpur, 29.84% Sonbhadra and 33.45% in Mirzapur); 97.06% of the population were Hindu; 50.5% were scheduled caste/scheduled tribe and 40.05% were other backward caste. The average age of the adolescent girls was 16.24 years (SD: 1.81) (not shown on table). On average, adolescent girls were in 9th grade (SD: 2.7) (not shown on table). For housing, a little less than half the adolescent girls (47.11%) lived in raw/temporary housing. About 99% of the adolescent girls were unmarried and 86.34% of the adolescent girls initiated menarche more than a year ago. For MHM behaviors: adequate preparation was 23.42%; adequate storage was 60.53%, adequate privacy to change was 53.12%, adequate frequency of changing was 63.79%, adequate disposal was 40.55% and adequate hygiene was 58.32%. Overall, 27.58% of the adolescent girls practiced all behaviors adequately (not shown on table).

**Table 4:** Socio-demographic, Socio-economic Characteristics, duration since menarche and MHM behaviors among Adolescent Girls in Rural Uttar Pradesh

Characteristics	Overall (%)
**N**	**2212**
**District**	
*Jaunpur*	36.71
*Sonebhadra*	29.84
*Mirzapur*	33.45
**Religion**	
*Non-Hindu*	2.94
*Hindu*	97.06
**Caste**	
*General Caste*	9.45
*Scheduled caste/tribe*	50.50
*Other Backward Caste*	40.05
**Type of House**	
*Kutcha (Raw/temporary)*	47.11
*Semi-Pucca (Frame is concrete, but walls are raw/temporary)*	24.46
*Pucca (Solid/Concrete)*	28.44
**Marital status**	
*Married*	0.86
*Unmarried*	98.87
*Other*	0.27
**Duration since menarche**	
*< 1 year*	13.66
≥1 *year*	86.34
**MHM indicators**	
*Adequate preparation of clean absorbent*	23.42
*Adequate storage of clean absorbent*	60.53
*Adequate privacy to change*	53.12
*Adequate frequency of changing*	63.79
*Adequate disposal*	40.55
*Adequate hygiene*	58.32

### Results from the quantitative exploratory factor analysis of MHM behaviors

Table 5 demonstrates a single factor from the Principal Component Analysis with an eigenvalue of 1.70 and variance of 84.47%. Only two items loaded adequately on the factor: adequate privacy to change and adequate hygiene, with factor loadings of 0.91 each. All the other four items loaded separately.

**Table 5:** Principal Component Analysis for MHM Scale during Endline

MHM behaviors	Factor 1
**N**	2212
**Eigenvalues**	1.70
**Variance explained**	84.47%
*Adequate preparation of clean absorbent*	0.16
*Adequate frequency of changing absorbent everyday*	0.08
*Adequate disposal*	0.03
*Adequate storage of clean absorbent*	0.16
*Adequate privacy to change absorbent*	0.91
*Adequate hygiene*	0.91

### Results from the qualitative FGDs: “Day in the Life of”

A total of 36 FGDs with 309 participants were conducted, with each FGD having anywhere from 6 to 12 adolescent girls. Thirty four percent of the adolescent girls were from Jaunpur and Sonebhadra, each and 31.39% were from Mirzapur. The average age of the adolescent girls was 16 years. Ninety one percent of the participants were Hindu and 92% were either scheduled caste/scheduled tribe or other backward caste. The average education of adolescent girls was 9th grade.

For the “Day in the life of” activity, MHM behaviors for the sanitary pad were assessed. About half of the FGDs stated that sanitary pads were used during menstruation and made out of cotton: “I’m used during menstruation, made out of cotton and buried or burned after usage”. Other FGDs outlined the ease and protective properties of a sanitary pad: “It is easier to use a pad”, “It feels good to use a pad” and “a pad keeps us protected”. Most of the FGDs stated the sanitary pad was bought from market/shop wrapped in paper/plastic whereas some specified “in a polythene bag/black plastic”. For storage, 78% of the FGDs mentioned storing the sanitary pad in a safe and clean place including a bag, other clothes or cupboard; however, some FGDs also said they kept the pad “hidden between other clothes and in the dustbin”. One FGD outlined “inside the room/house” but did not specify where exactly they stored the pad. In terms of how it is used, most (83%) of the FGDs stated that a sanitary pad is used by removing the paper/plastic/sticker and sticking it on the underwear. However, one FGD highlighted how a pad was “hidden and used secretly”. For changing the sanitary pad, 86% of the FGDs outlined toilet/bathroom and about a quarter mentioned inside the house or room. Some FGDs mentioned “I am changed behind a *tatri* (temporary structure)” or “I am changed discreetly”. However, whether the changing place was private, was not mentioned. Approximately, 65 % of the FGDs reported changing the absorbent three or more times in a day. When asked how a sanitary pad was carried around, a little less than half of the FGDs stated it should be carried around secretly, wrapped in “cloth/dupatta” or “wrapped in paper/newspaper”. Most FGDs outlined that the sanitary pad is done when it gets wet. Fifty six percent of the FGDs outlined that a sanitary pad should be disposed by burning or burying in a pit, whereas others said in a water body, dustbin or agricultural field.

“Day in the life of” cloth was also assessed. A little more than half the FGDs stated that the cloth was a “cloth used for menstruation”. One FGD outlined that “a cloth was inconvenient to use”. The most common material the cloth was made from was cotton and thread. Other FGDs outlined silk thread and taken from torn cloth. Most (75%) FGDs stated the cloth came from home or old clothes at home, others said from market/shop, factory or part of a washed old cloth. Seventy eight percent of the FGDs outlined that a cloth should be stored in a safe clean place and used by washing and folding the cloth. As with the sanitary pad, other places of storage included storage inside the room in the house. Eighty one percent of the FGDs stated that cloth should be changed in a toilet, bathroom or bathing area; Other places where the cloth was changed included “behind the bush” and “behind a temporary structure”. Sixty one percent outlined changing the cloth three or more times a day; Almost all FGDs stated that clothes could be reused between 1 and 3 months. Although most (72%) of the FGDs said a cloth absorbent should be washed with soap and water or in the bathroom/home and dried in the sun; the FGDs also elicited other methods including “washing with hot water and soda and drying the cloth in the shade, beneath other clothes in the sun or on the terrace in a hidden manner and inside the house”. When looking at the data of how a cloth should be carried around, an equal proportion (28%) stated carried around discreetly/secretly and in a polythene bag/plastic. Other forms of carrying the cloth included wrapped in paper/newspaper, wrapped in a scarf and in a purse/bag. As with the sanitary pad, almost all FGDs outlined that they were done using the cloth when it got wet, others mentioned when it got dirty and sticky. Lastly, a little more than half of the FGDs (61%) mentioned the correct disposal method of burning or burying in a pit but other methods included in a water body, dustbin and agricultural field.

The first underlying theme that emerged from the FGD was menstruation as a taboo. Adolescent girls mentioned getting the absorbent secretly/discreetly, hiding the absorbent when storing it (hidden between other clothes); drying it in the sunlight but hidden under other clothes or in a hidden manner; changing it discreetly and carrying it around secretly. Additionally, the FGDs demonstrated incorrect methods for disposal, via a water body (canal, pond, drain, near a well) (14%).

A second theme that emerged was the lack of privacy for changing the absorbent. When asked where the absorbent was changed, most adolescent girls mentioned inside the house, toilet/bathroom; but other FGDs mentioned behind a temporary structure and behind the bush. When comparing the FGDs proportions to the structured questionnaire; storage, privacy to change and disposal proportions were slightly higher in the FGDs in comparison to the structured questionnaire. Adequate frequency of changing corroborated with the structured questionnaire.

## Discussion

The objective of this manuscript was to develop and test a MHM scale using the joint monitoring program definition, review of literature and the framework developed by Muralidharan among post-menarche adolescent girls in rural Uttar Pradesh using a mixed method design. A draft version of the scale was pretested during the concurrent monitoring in October 2016 and six experts were consulted to provide feedback on the individual items in the scale prior to field work. A mixed method approach was taken to analyze the research question and enrich the findings. The structured questionnaire provided population level data to objectively assess MHM behaviors and conduct factor analysis of a new scale. The FGDs enriched the structured questionnaire findings by eliciting the theme of menstruation as a taboo, inadequate privacy to change and incorrect disposal via a water body. When triangulating the results between the structured questionnaire and FGDs, the behavior proportions for the FGDs were slightly higher than that of the structured questionnaire since the unit of analysis was the FGD.

Within the structured questionnaire, adequate preparation of clean absorbent, storage of clean absorbent, privacy to change, frequency of changing, disposal and hygiene had a prevalence of 23.42, 60.53, 53.12, 63.79, 40.55 and 58.32%, respectively. Preparation of clean absorbent as a construct has not been operationalized before. Adequate storage has ranged from 50 to 90% within literature [13, 25]. Privacy to change was 48.75% [23]; Adequate frequency of changing has ranged from 14.82–45.5% [19, 25]; Adequate disposal has ranged from 3.6–76% [10, 20, 22] and hygiene proportions have ranged from 35.4–85% [10, 22, 23, 25]. The proportions from this study are generally in the middle of the range cited within literature. These proportions suggest that MHM behaviors within these three districts are representative with regard to other studies in India [10, 13, 19, 20, 22, 23, 25].

The proportions found for the six indicators of MHM are low (< 65%) and comparable to those within literature in India. Interestingly, behaviors which require infrastructure or family support (preparation of clean absorbent, privacy to change, hygiene and disposal of absorbent) have lower proportions in comparison to behaviors which are personal decisions (storage of clean absorbent and frequency of changing absorbent). These findings highlight why adequate MHM behaviors should be looked though the socio-ecological framework. At the individual level, there is a need to improve information about adequate storage and frequency of changing the absorbent. At the family level, it is important to involve fathers and mothers to increase access to clean absorbents, disposal facilities, private place to change and facilities to maintain adequate hygiene within the home environment. Additionally, programs can educate and change attitudes among family members about restrictions and menstrual taboos which affect adequate MHM behaviors [32]. At the community level, frontline health workers can disseminate knowledge about correct MHM behaviors to adolescent girls and family members; and advocate for safe disposal facilities with community leaders.

The low proportions of adequate MHM behaviors and the FGDs suggest that menstruation is still considered a taboo and behaviors which are linked to it cannot be talked about. When asked how an absorbent was procured, stored, dried and carried, many FGDs stated it was stored and dried in a hidden manner and carried discretely/secretly. Literature has consistently demonstrated that menstruation and menstruation related behaviors are considered a taboo around the world and have a negative relationship on MHM behaviors [2, 4, 33, 34]. This calls for programs to create messaging which increases communication and dialogue within the community to make menstruation a normal experience.

Literature has outlined a definition for MHM [3, 4]. However, the results of this study show that MHM behaviors comprise of four independent menstrual health behaviors and hygiene management behaviors. The author recommends that MHM is considered a multi-dimensional construct which comprises of these two dimensions. Menstrual health indicators include preparation of clean absorbent, storage of clean absorbent, frequency of changing and disposal, which are time bound to menstruation. Hygiene management indicators include privacy to change and hygiene. Contrary to the menstrual health indicators, the hygiene management indicators are not time-bound to menstruation. Within the literature, almost all scales measuring MHM have questions about hygiene [9–12, 14, 15, 17–19, 21–25]. It is therefore important to distinguish that, although privacy to change and hygiene are not time bound during the days of menstruation as the four menstrual health indicators, they are critical to managing menstruation adequately.

The conceptualization of MHM based on this study’s results is different from the MHM definition, but mimic Muralidharan’s framework. Muralidharan, distinguishes hygiene behaviors from menstrual absorbent in her framework. Although Muralidharan’s framework does not include private place to change, the joint monitoring program definition includes this indicator [3]. Muralidharan also highlights the importance of health indicators, socio-economic indicators, knowledge, attitudes related to menstrual behaviors and physical infrastructure/facilities as important constructs comprising of menstrual practices which are not operationalized in this paper’s MHM scale [26]. It is however, important to keep in mind that several of Muralidharan’s indicators served as determinants of MHM behaviors. For example, correct knowledge is a precursor to behavior as are socio economic factors such as availability of appropriate infrastructure. Defining, conceptualizing and operationalizing is a circular and inter-related process to measure and understand health behaviors within the population [35]. Future studies should operationalize other constructs proposed in Muralidharan’s framework and determine the validity and reliability of MHM as a whole.

This study operationalized the six behavioral indicators outlined within the definition and Muralidharan’s framework and found two dimensions. Although this operationalization might limit the scope of MHM as a whole by only operationalizing menstrual behaviors within Muralidharan’s framework, this scale expands on current scales operationalizing MHM behaviors, which primarily measure adequate MHM through a single item scale operationalized by type of absorbent used [36]. The results from this study expand the scope of adequate behaviors by demonstrating that five questions need to be asked in order to understand menstrual behaviors. Menstrual behaviors comprise of two dimensions: menstrual health and hygiene management. Therefore, the author proposes the following definitions for the two dimensions: Menstrual health can be defined as “use of clean menstrual management material to absorb or collect blood that is stored in a safe, clean place and changed at least three times a day for the duration of the menstruation period and accessing facilities to ultimately dispose of used menstrual management materials”. Hygiene management can be defined as “access and use of private toilet/bathroom with soap and water to wash hands and have a bath during menstruation”. When combined, menstrual health and hygiene management (MHHM) can be defined as “Access and use of private toilet/bathroom with soap and water to wash hands and have a bath during menstruation and use of clean menstrual management material to absorb or collect blood that is stored in a safe, clean place and changed at least three times a day for the duration of the menstruation period and accessing facilities to ultimately dispose of used menstrual management materials”. Future studies should administer this scale and verify if this factor structure holds. The author will now use the acronym MHHM from this point forward.

Limitations of this study included lack of criterion validity, convergent and discriminant validity, predictive validity, reliability testing, scale limitations and generalizability. Due to the lack of validity and reliability tests on other scales in literature, comparison of this scale could not be done to assess criterion validity. Convergent and discriminant validity could not be tested because psychometric properties of other MHM scales within literature was not carried out to compare the factor structure. However, to understand construct validity, the author conducted factor analysis twice to see if the factor structure was upheld, conducted FGDs and got validation from an expert committee. Predictive validity could not be assessed because the evaluation was focusing on effectiveness rather than impact. Internal consistency could not be conducted because only two indicators loaded on a single factor. Test-retest reliability was not applicable because this was a new scale. Inter-rater reliability was not applicable because the scale was created and coded by the author. A limitation of the MHM scale included: 1) not considering individual variation in menstrual pattern, e.g. duration and amount of bleeding and 2) not addressing new menstrual absorbents i.e. menstrual cups. Individual variation in menstrual pattern was not included because it was not considered a MHM behaviour. New menstrual absorbents were not included in the scale because they were not cited as valid responses within the population during the pilot testing and during this study. Lastly, generalizability of these findings may be only applicable to Uttar Pradesh. However, most of the results found in this study were comparable to those within literature in India.

## Conclusions

MHM as a scale has not been operationalized and validated within literature. This study uses the joint monitoring program MHM definition, scales within literature and the framework developed by Muralidharan, to operationalize and carry out psychometric testing of a MHM scale among adolescent girls in rural Uttar Pradesh. Based on the validity testing of the scale, MHM is a multi-dimensional construct comprising of two dimensions: menstrual health and hygiene management. The author proposes a new definition for MHHM and recommends that future research within this realm should use the acronym MHHM rather than MHM and conduct further testing of this scale.

## Supplementary information

**Additional file 1 Appendix 1**: Types of Validity and Reliability Tests Being Conducted and Justification. **Appendix 2**: Menstrual Hygiene Management Scale used during the Pilot. **Appendix 3**: Menstrual Hygiene Management Scale. **Appendix 4:** “Day in the Life of Disposable Sanitary Napkin/Cloth”

## Data Availability

The datasets used and/or analysed during the current study are available from the corresponding author on reasonable request.

## Abbreviations

FGD: Focus Group Discussion
MHHM: Menstrual Health and Hygiene Management
MHM: Menstrual Hygiene Management
