# Supplementary material for: What are the psychometric properties of a menstrual hygiene management scale: a community-based cross-sectional study
Source: BMC Public Health. 2020 Apr 19;20:525. doi: 10.1186/s12889-020-08627-3 (PMC7168814; doi:10.1186/s12889-020-08627-3)
Supplement: Supplementary file 1 — Additional file 1 Appendix 1: Types of Validity and Reliability Tests Being Conducted and Justification. Appendix 2: Menstrual Hygiene Management Scale used during the Pilot. Appendix 3: Menstrual Hygiene Management Scale. Appendix 4: “Day in the Life of Disposable Sanitary Napkin/Cloth” [file 12889_2020_8627_MOESM1_ESM.docx]

## Appendix 1: Types of Validity and Reliability Tests Being Conducted and Justification

| Type of validity or reliability | Application to dissertation & justification |
| --- | --- |
| Content validity | 1. The scale was based on the MHM definition and adapted based on other scales within literature, which covered some indicators of MHM but not all.  2. The tools were reviewed by an external review board group from UNICEF. |
| Criterion validity | There is no existing gold standard scale to measure MHM, therefore this validity cannot be tested. |
| Construct validity | 1. Conducted factor analysis to show underlying unobservable variables. Compared the factor structure to the pilot, although items changed.  2. Qualitative focus group discussions were used to validate and triangulate the structured questionnaire.  3. Convergent and discriminant validity could not be tested because psychometric properties of other scales within literature was not carried out to compare/contrast. However, proportions were compared. |
| Predictive validity | Predictive validity of the MHM scale could not be assessed since the larger study was focusing on effectiveness rather than impact. |
| Internal consistency | Internal consistency could not be conducted because only two indicators loaded together in a single factor. The other indicators were separate constructs |
| Test-retest reliability | This was not applicable because a new scale was implemented. |
| Inter-rater reliability | This was not applicable because the scale was created and coded by one person. |

##

## Appendix 2: Menstrual Hygiene Management Scale used during the Pilot

| Question | Construct | Question | Responses | Adequate MHHM (1) | Inadequate MHHM (0) |
| --- | --- | --- | --- | --- | --- |
| 1 | Preparation of clean absorbent | What type of menstruation absorbent do you use?  *Multiple response* | 1. new cotton cloth every time I change  2. Old cotton cloth  3. Any other synthetic cloth  4. Disposable sanitary napkin/pad  5. Reusable sanitary napkin/pad | If option 1 or 4 selected in question 1 and question 1.1 or 1.2 not answered  OR  If option 2 and 5 selected in question 1, AND option 2 or 3 only selected in 1.1 and only option 3 selected in 1.2. | Any other responses which don’t match the correct MHHM behaviors. |
| 1.1 | Preparation of clean absorbent – Old cotton cloth (option 2) or reusable sanitary napkin/pad (option 5) only in question 1 | How do you wash your menstrual cloth?  *Multiple response* | 1. Water only  2. Soap and water  3. Any other disinfectant after washing with soap and water  4. Hot water |  |  |
| 1.2 | Preparation of clean absorbent – Old cotton cloth (option 2) or reusable sanitary napkin/pad (option 4) only in question 1 | Where do you dry your menstrual cloth?  *Multiple response* | 1. In the shade outside  2. In the shade inside  3. In the sunlight outside  4. Hidden under other clothes |  |  |
| 8 | Storage of clean absorbent | How do you store your menstrual absorbent?  *Multiple response* | 1. Store the absorbent in a hidden or concealed place  2. Store the washed menstrual cloth along with other clothes of daily wear  3. Bag  4.Does not store, takes new one every month  5. Store in a safe clean place  6. In cupboard  9.In a bag, hang it  10.In a plastic bag  11. Polythene bag  12.Wrapped in paper, kept in bag | If selected option 2, 5, 6 in question 8 |  |
| 7 | Ability to change absorbent in privacy | In your opinion, to what extent is the place where you change your absorbent most often, private? | 1. always  2. mostly  3. occasionally/sometimes  4. rarely  5. never | If selected option 1 in question 7 |  |
| 6 | Frequency of changing | How many times do you change your menstrual absorbent every day? | 1. never  2. 1 – 2 times a day  3. 3 or more times a day  4. as frequently as required | If selected option 3 in question 6 |  |
| 3 | Hygiene | During menstruation, do you take a bath daily with soap and water? | 1. Yes  2. No | If selected option 1 in question 3, 4, 5 AND 6. |  |
| 4 | Hygiene | While changing your menstrual absorbent, do you clean your genital area? | 1. Yes  2. No |  |  |
| 5 | Hygiene | Do you wash your hands with soap and water before cleaning your genital area? | 1. Yes  2. No |  |  |
| 6 | Hygiene | Do you wash your hands with soap and water after cleaning your genital area? | 1. Yes  2. No |  |  |
| 9 | Disposal | How do you dispose off your used menstrual absorbent? *Multiple response* | 1. Bury it in the soil in the field  2. Throw it in the bush  3. Burn the absorbent  4. Hide under a stone  5. Store and then take it to the school toilet incinerator  6. Dustbin or garbage lot  7. Bury in a pit  8. Pond water body  9. Gutter  10. Wash it | If selected option 3,5, or 7 only for question 9.  OR  If selected 2 or 5 in question 1 AND 1,2,3,4,5 in 9.1. AND selected 3,5,7 only for question 9. |  |
| 9.1 | Disposal – Old cotton cloth (option 2) or reusable sanitary napkin/pad (option 4) only in question 1 | How often do you dispose off your used menstrual cloth? | 1. Daily 2. Weekly   3. 1 month  4. 2 months  5. 3 months  6. ≥4 months |  |  |

**Appendix 3: Menstrual Hygiene Management Scale**

| Question | Construct | Question | Responses |
| --- | --- | --- | --- |
| 1 | Procurement of clean absorbent | What type of menstruation absorbent do you use?  *Multiple response* | 1. new cotton cloth every time I change  2. Old cotton cloth  3. Any other synthetic cloth  4. Disposable sanitary napkin/pad  99.. Any other (specify) ______________ |
| 1.1 | Procurement of clean absorbent – Old cotton cloth (option 2) | Do you wash the cloth before you use it to absorb menstrual blood? | 1. Always 2. Mostly 3. Sometimes/occasionally 4. Rarely   Never |
| 1.2 | Procurement of clean absorbent – Old cotton cloth (option 2) | Do you re-use the used menstrual cloth? | 1. Yes  2. No |
| 1.3 | Procurement of clean absorbent – Old cotton cloth (option 2) | How do you wash your menstrual cloth?  *Multiple response* | 1. Water only  2. Soap and water  3. Any other disinfectant after washing with soap and water  4. Hot water  99.. Any other (specify)______ |
| 1.4 | Procurement of clean absorbent – Old cotton cloth (option 2) | Where do you dry your menstrual cloth?  *Multiple response* | 1. In the shade outside  2. In the shade inside  3. In the sunlight outside  4. I hide it  99. Any other (specify) ________ |
| 1.5 | Procurement of clean absorbent – Old cotton cloth (option 2) | How often is soap available for washing of menstrual cloth? Would you say always, mostly, occasionally/sometimes, rarely or never | 1. Always 2. Mostly 3. Sometimes/occasionally 4. Rarely 5. Never |
| 1.6 | Procurement of clean absorbent – Old cotton cloth (option 2) | Do you use menstrual cloth exclusively or share with other female members of the family? | 1. Exclusively use 2. Shared with others |
| 2 | Storage of clean absorbent | How do you store your menstrual absorbent?  *Multiple response* | 1. Store the absorbent in a hidden or concealed place  2. Store the washed menstrual cloth along with other clothes of daily wear  3. Bag  4.Does not store, takes new one every month  5. Store in a safe clean place  6. In cupboard  7.In a bag, hang it  8.In a plastic bag  9. Polythene bag  10.Wrapped in paper, kept in bag  99. Any other (specify) ______________ |
| 3 | Ability to change absorbent in privacy | During menstruation, where do you change your absorbent? | 1. In a private bath area 2. In a private toilet 3. Behind a curtain 4. Behind a temporary structure |
| 4 | Frequency of changing | How many times do you change your menstrual absorbent every day? | insert numeric response |
| 5 | Hygiene | Is there a separate bathing place at home? | 1. Yes  2. No |
| 6 | Hygiene | Do you normally use it for bathing? | 1. Yes  2. No |
| 7 | Hygiene | Do you use the bathing area at home during menstruation? | 1. Yes  2. No  3. Do not bathe during menstruation |
| 8 | Hygiene | Do you normally take a bath with soap and water? | 1. Yes  2. No |
| 9 | Hygiene | During menstruation do you take a bath daily with soap and water? | 1. Yes  2. No |
| 10 | Hygiene | During menstruation, do you always wash your hands with after changing your menstrual absorbent? | 1. Yes  2. No |
| 11 | Hygiene | How do you wash your hands after changing your menstrual absorbent? | 1. With only water/ 2. With soap and water 3. With ash and water 4. With mud and water 5. Others (specify)/ |
| 12 | Disposal | How do you ultimately dispose of your used menstrual absorbent? *Multiple response* | 1. Bury it in the soil in the field  2. Throw it in the bush  3. Burn the absorbent  4. Hide under a stone  5. Store and then take it to the school toilet incinerator  6. Dustbin  7. Garbage lot  8. Bury in a pit  9. Pond water body  10. Gutter  11. Wash it  99. Any other (specify) _____________ |

## Appendix 4: “Day in the Life of Disposable Sanitary Napkin/Cloth”

For this next activity, let us split into two groups: (1) One group will represent the sanitary pad, and the other group (2) the cloth. Both groups will answer the same set of questions, only that one group will answer the questions from the perspective of the sanitary pad while the other from the point of view of the cloth. You have to imagine what the sanitary pad or cloth is experiencing during the various stages of the menstrual cycle.

1. Please describe me
2. What am I made of?
   1. Immediately before menstruation starts what are the things you do with me?
   2. Where do I come from?
   3. How do you get me?
   4. Where am I stored?
3. During menstruation what are the things you do with me?
4. How am I used?
5. Where am I changed?
6. How frequently am I changed?
7. Am I re –used?
8. How many months am I used for (Cloth only)?
9. How am I cleaned? Where am I dried? (Cloth only)
10. How do you carry me around?
11. How do you know when you are done using me?
12. What do you do with me once you are done with me?
13. After menstruation
14. Where am I ultimately disposed of?
15. How am I ultimately disposed of?
